# Dose-dependent QTc interval prolongation under haloperidol and pipamperone in the management of delirium in a naturalistic setting

**DOI:** 10.3389/fpsyt.2023.1257755

**Published:** 2023-10-03

**Authors:** Philipp Bohny, Soenke Boettger, Josef Jenewein

**Affiliations:** ^1^Center for Psychiatry and Psychotherapy, Triaplus Clinic Zugersee, Zug, Switzerland; ^2^Department of Consultation-Liaison-Psychiatry and Psychosomatic Medicine, University Hospital of Zurich, Zurich, Switzerland; ^3^Faculty of Medicine, University of Zurich, Zurich, Switzerland; ^4^Privatklinik Hohenegg, Meilen, Switzerland

**Keywords:** delirium, QTc interval, torsades de pointes, haloperidol, pipamperone

## Abstract

**Objective:**

Delirium is an acute, life-threatening neuropsychiatric disorder frequently occurring among hospitalized patients. Antipsychotic medications are often recommended for delirium management but are associated with cardiovascular risks. This study aimed to investigate the frequency and magnitude of QTc interval prolongation and clinically relevant side effects occurring in delirium patients managed with haloperidol and/or pipamperone.

**Methods:**

This descriptive retrospective cohort study evaluated 102 elderly (mean age: 73.2 years) inpatients with delirium treated with either haloperidol, pipamperone, a combination of both, or neither in a naturalistic setting over the course of up to 20 days or until the end of delirium.

**Results:**

A total of 86.3% of patients were treated with haloperidol and/or pipamperone at a mean daily haloperidol-equipotent dose of 1.2 ± 1 mg. Non-cardiovascular side effects were registered in 2.9% of all patients and correlated with higher scores on the Delirium Observation Screening Scale. They did not occur more frequently under antipsychotic treatment. The frequency of QTc interval prolongation was comparably common among all groups, but prolongation magnitude was higher under antipsychotic treatment. It was positively correlated with antipsychotic dosage and the total number of QTc interval-prolonging substances administered. Critical QTc interval prolongation was registered in 21.6% (*n* = 19) of patients in the group treated with antipsychotics compared to 14.3% (*n* = 2) of patients in the unmedicated group; however, the difference was not statistically significant. Polypharmacy was associated with a higher risk of critical QTc interval prolongation and increased mortality during delirium.

**Conclusion:**

Delirium treatment with haloperidol and/or pipamperone was not associated with a higher risk of QTc-interval prolongation in this naturalistic patient sample but was greater in magnitude and correlated with equipotent dosage and the number of QT interval-prolonging substances used. Polypharmacy was associated with higher mortality and increased risk of critical QTc prolongation.

## 1. Introduction

Delirium is an acute, potentially life-threatening neuropsychiatric disorder (1) that frequently occurs in hospitalized patients and is characterized by fluctuating vigilance, disorganized thinking, affective lability, vegetative disinhibition, and psychotic symptoms. It is categorized into three subtypes: hypoactive, dominated by apathy and a decrease in executive functions; hyperactive delirium with predominant restlessness and often aggressive behavior; and the mixed form of delirium in which characteristics of both conditions are present, changing within the course of hours. The complex pathophysiology of delirium involves various neurotransmitter systems (e.g., acetylcholine, glutamate, and gamma-aminobutyric acid) and is not fully understood (2). Estimated prevalence rates of delirium among medical inpatients vary between 11 and 42% (1), and it tends to be underdiagnosed, especially its hypoactive subtype (3). In intensive care unit (ICU) settings, the prevalence can easily exceed 80% (4). Correct diagnosis and consecutive management of delirium are crucial since its persistence is associated with prolonged hospitalizations, elevated risk for various complications, residual neurocognitive deficits, and increased mortality (5).

Treatment of delirium is a challenging issue and can be partially contradictory in its dilemma of providing optimal relief for suffering patients while also following the “Nihil nocere” principle of primarily not causing harm through therapy (6). Treatment guidelines for non-withdrawal delirium primarily recommend the elimination of underlying causes (e.g., anemia, electrolyte imbalance, infections, drug effects, and pain) and non-psychopharmacological strategies such as orientation aids, mobilization, optimization of circadian activation, involvement of family, or reduction of environmental stimuli (7). In addition, antipsychotics are frequently prescribed, especially if severe agitation and distress occur; however, their use correlates with increased mortality (8) and provides conflicting results on the duration and outcome of delirium (9, 10). Haloperidol is usually considered the established standard (7), although, in practice, other antipsychotics are frequently used as well (e.g., risperidone, pipamperone, and quetiapine) and have been found to be effective (11, 12). While some authors point out the delirogenic potential of benzodiazepines (13), a recent meta-analysis demonstrated better outcomes from the addition of lorazepam to haloperidol (1).

A common side effect of any antipsychotic pharmacotherapy is the prolongation of QTc interval. It is caused by the blocking of specific cardiac cellular hERG-potassium channels (14) and can lead to lethal ventricular arrhythmia (torsades de pointes), although the relation between the risk for torsades de pointes and the magnitude of QTc interval prolongation is not strictly linear (15), with the latter nonetheless being an important surrogate marker (16). Not all antipsychotics have the same pro-arrhythmic potential (14), and our knowledge about the relative cardiac risks under different combinations of antipsychotic drugs is still insufficient. In this study, we retrospectively evaluated QTc intervals and other possible therapy side effects among 102 cases of inpatients with delirium receiving either no pharmacotherapy, haloperidol, pipamperone, or both.

## 2. Materials and methods

### 2.1. Patients

In this retrospective, descriptive cohort study, 102 patients, who were treated at different departments of the University Hospital Zurich between March 1, 2012, and June 26, 2015, were selected from an ongoing delirium evaluation that was part of a larger research project. Inclusion criteria were adult age (18 years or older by the time of admission), a diagnosis of hyperactive, hypoactive, or mixed delirium, an existing electrocardiography (ECG) performed at baseline within 60 days before the onset of delirium (ECG_a_, Day-A), and a further ECG performed later during delirium (ECG_b_, Day-B). We included only patients who received no potentially QTc interval-prolonging substances (QIPSs) other than haloperidol and pipamperone on Day-B or who had an established therapy with any QIPS continued and unaltered between days a and b.

We used the CredibleMeds^®^ database (17) from the Arizona Center for Education and Research on Therapeutics (AZCERT) as a reference to determine QTc interval-prolonging potential of QIPSs.

### 2.2. Ethics approval and consent to participate

This study is part of a larger research project, which was approved by the Ethics Committee of the canton of Zurich, Switzerland (KEK-ZH-Nr: 2012-0263), and was carried out in accordance with the Declaration of Helsinki, considering local regulations and standards.

### 2.3. Definition of delirium

Delirium was defined by fulfilling its diagnostic criteria according to the tenth edition of the International Classification of Mental and Behavioral Disorders, ICD-10 (alterations of consciousness and attention span, cognitive decline, psychomotor change, sleep disturbances, and affective symptoms with typical circadian fluctuation) (18) and by scoring positively on the 13-item Delirium Observation Screening Scale (DOS) (19). From suspicion of incident delirium, DOS scores were measured by specifically trained nursing staff every 8 h, and delirium assessment was performed until the end of delirium, 20 days at maximum. The first day of delirium was defined as day 1. Persistence of delirium was assumed if the mean DOS score during that day exceeded 2, at least one DOS score during the day exceeded 3, and/or at least two DOS scores exceeded 2. Remission of delirium was defined as a continuous absence of delirium over 24 h. Single missing DOS scores were reconstructed by calculating the arithmetic means of their adjacent scores. We calculated the mean DOS score on day 1 (DOS_1_) and over the duration of delirium (DOS_mean_).

### 2.4. Delirium management

Antipsychotics were used (haloperidol and pipamperone), sometimes combined with various benzodiazepines (midazolam, lorazepam, diazepam, alprazolam, triazolam, oxazepam, bromazepam, and clonazepam), to manage delirium. All patients received standard non-pharmacological antidelirious therapy. Patients treated with haloperidol monotherapy were summarized as group H, those with pipamperone represented group P, and those receiving a combination of both represented group HP. Group N consisted of patients receiving neither haloperidol nor pipamperone. Additional benzodiazepines were used in patients of all groups with comparable incidence (*p* = 0.978).

Haloperidol equipotency (1 eH $\hat{=}$ 1 mg/d haloperidol $\hat{\approx }$ 50 mg/d pipamperone) was estimated and calculated for pipamperon to compare dosages (20). Exposure to antipsychotics was formally assumed for eH ≥ 0.02.

### 2.5. Electrocardiography

In cases where multiple ECGs were performed within 60 days before delirium, the one conducted closest to day 1 and under the least exposure to antipsychotics and any QIPS was selected as ECG_a_. To determine the latter, we estimated plasma concentrations for all drugs involved based on a pharmacokinetic model with approximated half-life durations. QTc intervals on days a and b (QTc_a_, QTc_b_) were measured both automatically and manually, with excellent accordance between the measurements (r > 0.999, *p* < 0.001), applying the widely used Bazett correction formula. The difference between QTc_a_ and QTc_b_ was calculated for all patients (Δ_QTc_). In cases where more than one ECG was performed during delirium, the one conducted under the highest exposure to antipsychotic pharmacotherapy was chosen for ECG_b_.

Applying the limits suggested by the American Heart Association, we defined QTc intervals of >470 ms for men and > 480 ms for women (15) as critical QTc interval prolongations (CQPs).

### 2.6. Statistical methods

All analyses were performed using the IBM Statistical Package for Social Sciences (SPSS), version 20/25. We calculated means, standard deviations, medians, and interquartile ranges (IQRs) for descriptive statistics (QTc intervals, age of patients, day of ECG, dosages of medications, numbers of QIPS, etc.). T-tests (Mann–Whitney tests for non-parametric data) were used to compare means between the treatment subgroups. Analyses of variance (Kruskal-Wallis tests for non-parametric data) with post-hoc Bonferroni correction were performed for multiple comparisons. Pearson χ^2^ tests were applied to detect correlations between non-nominal variables. In samples of *n* < 20, we used Fisher’s exact test. The Kolmogorov–Smirnov method was applied to test for normal distribution. Subgroups of *n* < 10 were excluded from statistical analysis. A probability value of *p* < 0.05 was defined as the level of statistical significance.

## 3. Results

### 3.1. Patients characteristics

Of all patients, 72 patients (70.6%) were male, and 30 patients (29.4%) were female, with a median (IQR) age of 74.9 (11.4) years (Table 1). Most of them were treated at the departments of cardiology/cardiac surgery (44.1%), neurology/neurosurgery (22.5%), or angiology/vascular surgery (11.8%). A total of 13.7% of patients were under treatment with one or more QIPSs, mostly antidepressants (leading: citalopram, trazodone). Thirty-six patients (35.3%) were pre-diagnosed with at least one documented psychiatric disorder (most of them were organic), followed by substance use-related and mood disorders. Seventy-eight patients (76.5%) also had a cardiological diagnosis.

**Table 1:** Overview of socioepidemiological patient parameters and baseline ECG.

	No medication [N] (*n* = 14)	Any medication (*n* = 88)	Value of *p*	Haloperidol [H] (*n* = 10)	Pipamperone [P] (*n* = 43)	Haloperidol and pipamperone [HP] (*n* = 35)	Value of *p*^Ƹ^	Development of CQP under medication (*n* = 19)	No development of CQP under medication (*n* = 69)	Value of *p*^Ƹ^	All patients (*n* = 102)
Age, years^*^	68.3 (43.4–91, 15.8) [69.1, 26.7]	73.9 (41.2–100.6, 9.9) [75.2, 10]	0.239^£^	69.8 (41.2–86.8, 16.5) [73.9, 33.2]	74.3 (50.2–90.3, 8.7) [74.5, 9.8]	74.7 (54.6–100.6, 9) [75.3, 8.5]	0.414^†^ (0.659^†^)	76.3 (57–87.3, 8.2) [78.2, 13.7]	73.3 (41.2–100.6, 10.3) [74.6, 9.4]	0.222^£^ (0.243^†^)	73.2 (41.2–100.6, 11) [74.9, 11.4]
Gender, %											
Male	57.1	72.7	0.342^§^	60	74.4	74.3	0.654^§^ (0.493^§^)	84.2	69.6	0.204^§^ (0.222^§^)	70.6
Female	42.9	27.3		40	25.6	25.7		15.8	30.4		29.4
Psychiatric diagnosis, %	64.3	30.7	0.031^§^	30	34.9	25.7	0.682^§^ (0.096^§^)	31.6	30.4	0.924^§^ (0.061^§^)	35.3
Cardiac diagnosis, %	64.3	78.4	0.304^§^	70	81.4	77.1	0.666^§^ (0.531^§^)	68.4	81.2	0.344^§^ (0.238^§^)	76.5
DOS_1_^*^	5.4 (2.3–9, 2.2) [5.3, 3.3]	4.7 (1–13, 2.2) [4.5, 3]	0.249^∂^	3.9 (2–7, 1.5) [3.8, 2.3]	4.4 (1–11, 2.1) [4, 3]	5.2 (1.7–13, 2.4) [5, 2.7]	0.232^‡^ (0.135^‡^)	4.7 (2–8, 1.9) [5, 3]	4.6 (1–13, 2.3) [4.3, 3]	0.88^∂^ (0.511^‡^)	4.8 (1–13, 2.2) [4.7, 3.1]
Day of ECG_a_^*^	−3.6 (−15–-1, 4.2) [−2, 3]	−7.5 (−58–-1, 10.5) [−4, 6]	0.175^∂^	−7.2 (−36–-1, 10.5) [−4, 7]	−6.8 (−59–-1, 11.2) [−3, 5]	−8.5 (−37–-1, 9.7) [−5, 8]	0.483^‡^ (0.504^‡^)	−10.9 (−37–-1, 11.7) [−7, 14]	−6.6 (−59–-1, 10) [−3, 5]	0.113^∂^ (0.097^‡^)	−7 (−59–-1, 9.9) [−3, 6]
QTc_a_, ms^*^	438.9 (398–499, 27.7) [439, 33]	440.2 (360–525, 36.6) [439, 53]	0.895^∂^	454.7 (393–525, 38.3) [453, 52]	441 (369–521, 36.5) [440, 54]	435.1 (360–508, 36.1) [429, 50]	0.461^‡^ (0.493^‡^)	447.8 (369–505, 37.4) [460, 52]	438.1 (360–525, 36.3) [434, 48]	0.306^∂^ (0.568^‡^)	440 (360–525, 35.4) [439, 50]
Pt with QIPS, %	21.4	12.5	0.403^§^	10	11.6	14.3	0.899^§^ (0.786^§^)	10.5	13	1^§^ (0.682^§^)	13.7
Number of QIPS, if any^*^	2.3 (1–4, 1.5) [2, −]	1.2 (1–2, 0.4) [1, 0]	0.077^£^	1 (1–1, 0) [1, −]	1.2 (1–2, 0.4) [1, 1]	1.2 (1–2, 0.4) [1, 1]	0.88^†^ (0.353^†^)	1 (1–1, 0) [1, −]	1.2 (1–2, 0.4) [1, 1]	0.727^£^ (0.18^†^)	1.4 (1–4, 0.9) [1, 1]
Department of admission, %											
Cardiology/cardiac surgery	35.7	45.5	0.197^§^	49	48.8	42.9	0.281^§^ (0.276^†^)	47.4	44.9	0.247^§^ (0.166^†^)	44.1
Angiology/vascular surgery	0	19.3		20	20.9	17.1		21.1	18.8		22.5
Neurology/neurosurgery	42.9	13.6		0	11.6	20		26.3	10.1		11.8
Other	21.4	21.6		31	18.7	20		5.2	26.2		21.6

^*^Mean (range, standard deviation) [median, interquartile range]. DOS_1_, Average DOS score on day 1; ECG_a_, ECG on Day-A at baseline; QTc_a_, QTc interval on Day-A; QIPS, QTc interval prolonging substance (other than haloperidol or pipamperone); CQP, Critical QTc interval prolongation. ^†^Kruskal-Wallis H test; ^‡^Analysis of variance (ANOVA); ^£^Mann–Whitney U test; ^§^Pearson-χ^2^ test; ^∂^T test. ^Ƹ^Top value of *p*: comparisons among subgroups; bottom value of *p* (in brackets): comparison including group N.

### 3.2. Specifications of delirium

The mean DOS_1_ of delirium was 4.8 (2.2), DOS_mean_ was 4.4 (1.6), and the median observation duration was 5 (7) days (Tables 1, 2). Remission was observed in 73.5% of all patients within 3 (5) days after delirium onset. The results for remission rates and duration of delirium were comparable among all groups.

**Table 2:** Overview of adverse effects and relevant QTc interval changes among treatment groups.

	No medication [*N*] (*n* = 14)	Any medication (*n* = 88)	Value of *p*	Haloperidol [H] (*n* = 10)	Pipamperone [P] (*n* = 43)	Haloperidol and pipamperone [HP] (*n* = 35)	Value of *p*^Ƹ^	Development of CQP under medication (*n* = 19)	No development of CQP under medication (*n* = 69)	Value of *p*^Ƹ^	All patients (*n* = 102)
DOS_mean_^*^	4.4 (2.3–7.1, 1.6) [4, 2.8]	4.4 (0.9–8.9, 1.7) [4.5, 1.9]	0.871^∂^	4.1 (1.5–7, 1.6) [3.9, 2.2]	4 (0.9–8.1, 1.5) [4.3, 1.8]	4.9 (1.7–8.9, 1.8) [4.7, 2.8]	0.146^‡^ (0.134^‡^)	4.8 (2.7–6.9, 1.2) [4.8, 1.5]	4.3 (0.9–8.9, 1.8) [4.3, 1.9]	0.212^∂^ (0.449^‡^)	4.4 (0.9–8.9, 1.6) [4.5, 1.9]
Day of ECG_b_^*^	3.6 (1–9, 3) [2, 4]	4 (1–19, 3.5) [3, 5]	0.683^∂^	5.5 (1–15, 5.7) [3, 12]	3.9 (1–19, 3.8) [2, 5]	3.7 (1–8, 2) [3, 3]	0.459^‡^ (0.484^‡^)	3.4 (1–8, 2.2) [2, 3]	4.1 (1–19, 3.8) [3, 5]	0.438^∂^ (0.672^‡^)	3.9 (1–19, 3.4) [3, 4]
Days from ECG_a_ to ECG_b_^*^	7.2 (3–23, 6) [5, 9]	11.5 (2–60, 11.5) [8, 7]	0.176^∂^	12.7 (2–49, 14.3) [7, 15]	10.7 (2–60, 12.5) [7, 7]	12.1 (2–38, 9.5) [9, 7]	0.819^‡^ (0.521^‡^)	13.7 (2–43, 11.4) [9, 13]	10.2 (2–60, 10.8) [8, 7]	0.23^∂^ (0.18^‡^)	10.9 (2–60, 11) [8, 8]
QTc_b_, ms^*^	438.5 (402–484, 28.9) [430.5, 57]	455.8 (394–516, 29.5) [449.5, 43]	0.043^∂^	452 (413–488, 25.4) [446.5, 46]	456.2 (394–514, 30.2) [450, 46]	456.5 (416–516, 30.3) [446, 47]	0.63^‡^ (0.238^‡^)	495.1 (471–516, 14.2) [493, 23]	445 (394–513, 22.5) [443, 33]	< 0.001^∂^ (< 0.001^‡^)	453.4 (394–516, 29.9) [448.5, 45]
Δ_QTc_, ms^*^	−0.4 (−80–62, 42.9) [9, 66]	15.6 (−77–141, 39.3) [10.5, 42]	0.166^∂^	−2.7 (−77–44, 36.1) [−0.5, 44]	15.2 (−48–141, 35.3) [10, 34]	21.3 (−56–118, 43.9) [16, 53]	0.235^‡^ (0.192^‡^)	47.2 (3–141, 42.4) [28, 73]	6.9 (−77–101, 33.8) [4, 40]	< 0.001^∂^ (< 0.001^‡^)	13.4 (−80–141, 39.9) [10.5, 42]
Pt with positive Δ_QTc_, %	57.1	65.9	0.556^§^	50	69.8	65.7	0.478 ^§^ (0.599^§^)	100	56.5	< 0.001^§^ (< 0.001^§^)	64.7
Mean dosages per day Day-B: ^*^ – eH	–	1.4 (0.1–4.5, 1) [1.2, 0.9]	–^£^	1.4 (0.3–3.4, 1.2) [1.2, 2.3]	0.8 (0.1–2.2, 0.5) [0.7, 0.7]	2.1 (0.4–4.5, 1) [2, 1.4]	< 0.001^†^ (−^†^)	1.6 (0.1–3.6, 1) [1.6, 1.5]	1.3 (0.1–4.5, 1) [1.1, 1.4]	0.241^£^ (−^†^)	1.2 (0–4.5, 1) [1, 1.5]
ADR, % (*n*): – any	7.1 (*n* = 1)	2.3 (*n* = 2)	0.361^§^	0	0	5.7 (*n* = 2)	0.373^§^ (0.26^§^)	0	2.9 (*n* = 2)	1^§^ (0.435^§^)	2.9 (*n* = 3)
Cardiovascular	0	0	–^§^	0	0	0	–^§^ (−^§^)	0	0	–^§^ (−^§^)	0
Death during delirium, %: – all	7.1	2.3	0.361^§^	10	0	2.9	0.102^§^ (0.103^§^)	0	2.9	1^§^ (0.435^§^)	2.9
Development of CQP, % (*n*)	14.3 (*n* = 2)	21.6 (*n* = 19)	0.729^§^	10 (*n* = 1)	20.9 (*n* = 9)	25.7 (*n* = 9)	0.625^§^ (0.734^§^)	100 (*n* = 19)	0	–^§^ (−^§^)	20.6

^*^Mean (range, standard deviation) [median, interquartile range]. DOS_mean_, Average DOS score over delirium; ECG_a_, ECG on Day-A at baseline; ECG_b_, ECG on Day-B during delirium; QTc_b_, QTc interval on Day-B; Δ_QTc_, Change in QTc interval from day 0 to day x; eH, Haloperidol equipotency (≙ 1 mg/d); ADR, Adverse drug reaction; CQP, Critical QTc interval prolongation. ^†^Kruskal-Wallis H test; ^‡^Analysis of variance (ANOVA); ^£^Mann–Whitney U test; ^§^Pearson-χ^2^ test; ^∂^T test. ^Ƹ^Top value of *p*: Comparisons among subgroups; bottom value of *p* (in brackets): Comparison including group N.

### 3.3. Pharmacological treatment of delirium: overview

Eighty-eight patients (86.3%) were under antidelirious pharmacotherapy with haloperidol, pipamperone, or both on Day-B (Table 2). Among those patients, 10 patients (11.4%) received haloperidol monotherapy, 43 patients (48.9%) received pipamperone monotherapy, and 35 patients (39.8%) received haloperidol combined with pipamperone. Pipamperone was the overall most frequently administered antipsychotic (88.6%).

Antipsychotic dosages on Day-B varied (*p* < 0.001) between eH = 0.1 and 4.5 (mean 1.2 ± 1) and were lowest in group P (0.8 ± 0.5) and highest in group HP (2.1 ± 1). DOS_1_ scores showed a tendency to correlate positively with eH on Day-B (r_P_ = 0.188, *p* = 0.08).

Patients receiving antipsychotics were more likely to have been previously diagnosed with a psychiatric disorder (*p* = 0.031). There were no significant differences in gender, age, pre-morbidity, number of QIPS administered, days between ECG_a_ and ECG_b_, or QTc interval at baseline between the treatment groups.

### 3.4. Adverse effects and fatal outcomes

In total, adverse drug reactions (ADRs) occurred in three patients (2.9%) on average on day 2.7 ± 0.6 of delirium (Table 2). All ADRs were non-cardiovascular (hypopnea, aspiration, and somnolence), and no case of torsades de pointes was registered. ADRs occurred in delirium with higher DOS_mean_ (*p* = 0.014, r_P_ = 0.243), and their manifestation appeared to be positively correlated with the length of QTc interval on Day-A (*p* = 0.026, r_P_ = 0.221). One case of ADRs (7.1%) was registered in group N.

Among patients receiving antipsychotics, all ADRs occurred in group HP. No significant correlation to the use of any specific substance nor the dosage was found. One 87-year-old polymorbid patient (1.1% of medicated patients) died from aspiration under a cumulative dose of 1 mg haloperidol and 90 mg pipamperone on day 6 of delirium (1.1 eH).

The total number of QIPSs prescribed correlated with the risk of dying during delirium from any cause (*p* = 0.005, r_P_ = 0.275).

### 3.5. Findings in ECG: overview

Across all patients, ECG_a_ was performed at a median of 9.9 (6) days prior to delirium onset, and the mean QTc_a_ was 440 ± 35.4 ms (Tables 1, 2). In 18 patients (17.6%), prolonged QTc interval was found in ECG_a_. ECG_b_ was performed after a median of 3.4 (4) days into delirium. The time between ECG_a_ and ECG_b_ (10.9 ± 11 days) did not differ significantly across treatment groups.

### 3.6. Findings in ECG: Δ_QTc_ and CQP under therapy

Across all patients, QTc increased by a mean of 13.4 ± 39.9 ms from Day-A to Day-B (QTc_b_ 453.4 ± 29.9 ms) (Table 2).

Among patients receiving any antipsychotics, 58 patients (65.9%) showed a positive Δ_QTc_, while only 8 patients (57.1%) showed a positive Δ_QTc_ in group N. Δ_QTc_ was −2.7 ± 36.1 ms in group H, 15.2 ± 35.3 ms in group P, and 21.3 ± 43.9 ms in group HP; however, these Δ_QTc_ differences between the treatment regimens and compared to group N were not significant. In group HP, dosages above the mean correlated with higher Δ_QTc_ (30.6 ± 46.5 ms vs. −2 ± 26 ms; *p* = 0.045). Overall, we found a negative correlation between Δ_QTc_ and the incidence of ADRs (r_P_ = −0.234, *p* = 0.018).

The mean QTc_b_ in patients receiving antipsychotics was 455.8 ± 29.5 ms (Δ_QTc_ 15.6 ± 39.3 ms) compared to 438.5 ± 28.9 ms (Δ_QTc_ − 0.4 ± 42.9 ms) in group N (*p* = 0.043). QTc_b_ of patients in groups P (456.2 ± 30.2 ms; *p* = 0.06) and HP (456.5 ± 30.3 ms; *p* = 0.064) showed a clear tendency to be longer compared to those in group N (438.5 ± 28.9 ms). The total number of QIPSs (haloperidol and pipamperone) administered tended to correlate positively with the length of QTc_b_ (r_P_ = 0.182, *p* = 0.067). Overall, each additionally administered eH predictively prolonged QTc_b_ by approximately +5.7 ms (*F*[1, 100] = 4.133, *p* = 0.045; R^2^ = 3%).

The rate of CQP among all patients was 20.6%, occurring after a median time of 2 (3) days. Nineteen patients (21.6%) with antipsychotics newly developed CQP on Day-B (mean 495.1 ± 14.2 ms); however, there was no significant difference with two patients in group N (14.3%, 478.1 ± 8.5 ms). The incidence of CQP did not differ significantly between groups H (10%), P (20.9%), and HP (25.7%). In patients receiving haloperidol plus pipamperone combination therapy, dosages above the mean correlated with a higher rate of CQP (36% vs. 0%; *χ*^2^[1] = 4.8, *p* = 0.036). Furthermore, no clear dose dependency on the risk of developing CQP was found in any group. The manifestation of CQP did not correlate with the incidence of ADRs.

## 4. Discussion

### 4.1. Summary of main findings

Pharmacological delirium management with antipsychotics, especially pipamperone, was very common (86.3%). Generally, antipsychotic dosages administered were low (mean eH on Day-B = 1.2 ± 1), and the use of combination therapy with haloperidol and pipamperone was correlated with higher eH on Day-B (2.1 ± 1).

ADRs were rare (2.9%) and mainly non-cardiovascular and occurred in patients receiving haloperidol plus pipamperone combination therapy. The probability of ADRs correlated positively with higher DOS scores and the length of QTc interval at baseline. Polypharmacy with multiple QIPSs was correlated with higher mortality during delirium.

QTc interval prolongation on Day-B was very common in all groups (haloperidol: 50%; pipamperone: 69.8%; haloperidol plus pipamperone: 65.7%; no antipsychotics: 57.1%) and was positively correlated with the dosages administered, especially under combination therapy with haloperidol and pipamperone. Treatment with antipsychotics *per se* was not associated with a higher incidence of QTc interval prolongation, but if prolongation did occur, it was 17.3 ms longer than in patients without haloperidol and/or pipamperone. The length of QTc interval on Day-B was positively correlated with the total number of QIPSs at baseline. The use of pipamperone monotherapy and haloperidol plus pipamperone combination, but not haloperidol monotherapy, tended to be associated with positive Δ_QTc_ on Day-B.

Overall, CQP was common (20.6%), and its occurrence did not significantly differ between the groups. Combination therapy with haloperidol and pipamperone dose-dependently increased the risk of CQP.

### 4.2. Interpretation

The finding that particularly the haloperidol plus pipamperone combination therapy was dose-dependently associated with an increased risk of CQP provides further evidence to favor monotherapy over polypharmacy whenever possible. However, in line with the existing literature (21), we found that the use of combination therapy was accompanied by higher equipotent antipsychotic dosages, which is suspected to be the main reason for the QTc interval-prolonging effect in polypharmacy with antipsychotics. The design of our study, the number of patients, and the low drug dosages used do not allow for any causal attribution, but further research on the complex interactions between antipsychotic polypharmacy, equipotent dosages, and QTc interval changes could provide clarification.

The use of pipamperone, which, in the light of its proven antidelirious property (12) and the *de facto* lack of anticholinergic effects, is often used to treat geriatric patients, was associated with an increase in QTc interval length. Despite the descriptive intent of this study, this finding supports the recommendation for frequent ECG control under pipamperone administration, particularly in vulnerable patient populations such as the one in this study.

The unexpected result that haloperidol monotherapy *per se* was not associated with the prolongation of QTc interval might be due to the low dosages used in the study. In addition to that, the mean QTc interval length at baseline in the haloperidol group was rather high (454.7 ± 38.3 ms), which might indicate a distorting effect of the QT interval correction method used. The Bazett formula is known to slightly overestimate elevated heart rates (22), by which patients with tachycardia of any etiology (anemia, infection, etc.) might have been diagnosed with baseline QTc interval prolongation, which, as a consequence, would falsely reduce Δ_QTc_.

The number of QIPSs was positively associated with Δ_QTc_ in the naturalistic setting of this study, indicating the importance of carefully considering all, not only psychotropic, patient’s medications when conceptualizing antidelirious pharmacotherapy and assessing for potential risks.

We further found that the risk of ADRs was particularly present in delirium with higher DOS scores, suggesting the need for close clinical monitoring and increased caution in the administration of pharmacotherapy to this vulnerable group. We interpreted the number of QIPSs as an indirect approximative indicator for global illness severity, which could explain its positive correlation to increased mortality during delirium.

The high rate of ADRs in the group of patients not receiving any antipsychotics was surprising at first. However, many of these patients were treated with benzodiazepines. Hence, we hypothesize the latter to be the reason for therapy-related side effects, as their associated potential risk is well documented (e.g., fall hazard, anticholinergic potential, paradoxical effects, and respiratory depression) (23).

Besides being a possible manifestation of hypoactive delirium *per se* (9), the occurrence of somnolence and sedation under combination therapy with haloperidol and pipamperone was not unexpected, as these symptoms are common under antipsychotic medication. Pipamperone is more strongly associated with somnolence and sedation than haloperidol (24) and clinicians should carefully consider these effects in their assessment.

In line with this hypothesis, we found a concomitant association between QTc interval length at baseline and ADR risk, which we believe is explained by a tendency of physicians to be less likely to prescribe antipsychotics for patients with long QTc intervals and rather use substances with less pro-arrhythmic potential (e.g., benzodiazepines).

Similarly to ADRs, the finding that QTc prolongation occurred frequently in patients in group N was unexpected. A possible reason could be the administration of benzodiazepines, as it was previously shown that benzodiazepines are not fully electrophysiologically inert to the heart. Diazepam, for example, can prolong the QTc interval (25), and midazolam might have some inhibitory hERG-channel affinity (26). In this context, one possible explanation could be that the application of benzodiazepines to patients in group N, especially intravenous midazolam in high dosages, might have induced QTc prolonging effects. Another reason could be distortion effects due to the use of the Bazett correction formula. Future research with a focus on prospectively evaluating QTc interval under benzodiazepine therapy and with rigorous control of potentially confounding factors could further clarify this question.

### 4.3. Limitations

Although the total number of patients included in this study was reasonably large, the size of the subgroups became small, limiting their statistical potential. Additionally, in this descriptive presentation of a naturalistic patient sample, the median age was rather high, and men were overrepresented, limiting generalizability.

Due to the retrospective study design, a variety of known risk factors for QTc interval prolongation (e.g., kidney failure, obesity, hepatic dysfunction, and malnutrition) could not be adequately considered in our calculations, and observed associations are not to be conflated with causal relationships. Also, the potential influence of autonomic dysregulation due to delirium itself or its effects on the QTc interval (27) has not been specifically evaluated.

Although the prescription rate of benzodiazepines did not significantly differ between the four groups, the dosages given did, relativizing comparability with regard to therapy adverse effects of pharmacotherapy.

Another limitation is that baseline and control ECGs were not performed at standardized time points in all patients. Therefore, the results and interpersonal comparisons need to be interpreted cautiously due to regular QTc interval dynamics over time.

Concerning the method for QT interval correction, the application of the Bazett formula might potentially overcorrect and lead to false-positive findings of (critical) QTc interval prolongation. This effect is known to be particularly relevant in patients with higher heart rates (22), which is frequently prevalent in stressful situations such as delirium. Also, the findings of this study mainly rely on the application of the QTc interval length limits suggested by the American Heart Association (see 2.5. Electrocardiography) and it is to be mentioned, that some authors recommend lower limits (28).

### 4.4. Conclusion

QTc-interval prolongation under haloperidol and/or pipamperone treatment of delirium in this naturalistic patient sample was not more frequent but greater in magnitude compared to patients not receiving antipsychotics. It was correlated with the dosage and the total number of administered QIPSs. Combination therapy was associated with higher mortality during delirium and a dose-dependent increase in the risk for CQP. ADRs were predicted by DOS scores and QTc-interval length at baseline. In summary, these results support the use of carefully dosed haloperidol and/or pipamperone pharmacotherapy in elderly inpatients suffering from non-withdrawal-associated delirium. Although QTc-interval prolongation is very common, treatment with antipsychotics is relatively safe regarding ADRs and critical QTc-interval alterations.

## Data availability statement

The raw data supporting the conclusions of this article will be made available by the authors, without undue reservation.

## Ethics statement

The studies involving humans were approved by Ethics Committee of the canton of Zurich, Switzerland (KEK-ZH-Nr: 2012-0263). The studies were conducted in accordance with the local legislation and institutional requirements. The participants provided their written informed consent to participate in this study.

## Author contributions

PB: Formal analysis, Investigation, Writing – original draft. SB: Conceptualization, Supervision, Writing – review & editing. JJ: Conceptualization, Methodology, Supervision, Writing – review & editing.

## Abbreviations

ECG_a_ (ECG_b_): Electrocardiography performed on Day-A (B)
DOS_1_: Delirium Observation Scale score assessed on day 1
DOS_mean_: Mean DOS score over the duration of delirium
QIPS: QTc interval-prolonging substances
eH: Haloperidol equipotency (1 eH ≙ 1 mg/d haloperidol)
CQP: Critical QTc interval prolongation
ADR: Adverse drug reactions
QTc_a_ (QTc_b_): Length of QTc interval on Day-A (B)
Δ_QTc_: Difference between QTc_a_ and QTc_b_
Group H: Patients treated with haloperidol
Group P: Patients treated with pipamperone
Group HP: Patients treated with haloperidol and pipamperone
Group N: Patients treated with neither haloperidol nor pipamperone
